# Flavivirus Concentrates Host ER in Main Replication Compartments to Facilitate Replication

**DOI:** 10.1002/advs.202305093

**Published:** 2023-10-27

**Authors:** Yali Ci, Kai Han, Jie Kong, Shuhan Huang, Yang Yang, Cheng‐Feng Qin, Lei Shi

**Affiliations:** ^1^ State Key Laboratory of Common Mechanism Research for Major Diseases Institute of Basic Medical Sciences Chinese Academy of Medical Sciences and School of Basic Medicine Peking Union Medical College Beijing 100005 China; ^2^ Department of Biochemistry and Molecular Biology Institute of Basic Medical Sciences Chinese Academy of Medical Sciences and School of Basic Medicine Peking Union Medical College Beijing 100005 China; ^3^ State Key Laboratory of Pathogen and Biosecurity Beijing Institute of Microbiology and Epidemiology Beijing 100071 China

**Keywords:** ER reorganization, flavivirus, NS proteins, replication compartments, superresolution live‐cell microscopy

## Abstract

Flavivirus remodels the host endoplasmic reticulum (ER) to generate replication compartments (RCs) as the fundamental structures to accommodate viral replication. Here, a centralized replication mode of flavivirus is reported, i.e., flavivirus concentrates host ER in perinuclear main replication compartments (MRCs) for efficient replication. Superresolution live‐cell imaging demonstrated that flavivirus MRCs formed via a series of events, including multisite ER clustering, growth and merging of ER clusters, directional movement, and convergence in the perinuclear region. The dynamic activities of viral RCs are driven by nonstructural (NS) proteins and are independent of microtubules and actin. Moreover, disrupting MRCs formation by small molecule compounds inhibited flavivirus replication. Overall, the findings reveal unprecedented insight into dynamic ER reorganization by flavivirus and identify a new inhibition strategy.

## Graphical abstract

Flavivirus adopts a centralized replication mode, i.e., flavivirus replicates in perinuclear main replication compartments (MRCs) derived from the endoplasmic reticulum (ER). Dynamic ZIKV MRCs formation is visualized by superresolution microscopy, including multi‐site ER clustering, the growth and merging of ER clusters, and directional movement toward the nucleus and convergence in the perinuclear region.

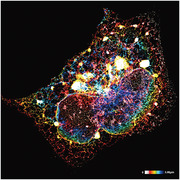

## 1 Introduction

Both DNA and RNA viruses establish replication compartments (RCs, or replication organelles/factories) to create environments that facilitate viral genome replication.^[^ ^1^, ^2^ ^]^ Many RNA viruses remodel cellular organelles to generate membranous vesicles as viral RCs for efficient viral replication and evasion of host immune recognition and intervention.^[3^ ^]^ The endoplasmic reticulum (ER), the central hub of protein and lipid biosynthesis in the cell, is often hijacked by positive‐strand RNA viruses, such as flavivirus and coronavirus, for the construction of viral RCs.^[^ ^4^, ^5^, ^6^ ^]^ Flavivirus, including Zika virus (ZIKV), dengue virus (DENV), and West Nile virus (WNV), poses serious threats to human health.^[7^ ^]^ Flavivirus genomic RNA is synthesized in membranous vesicular structures derived from the ER, i.e., flavivirus RCs. The typical structures of flavivirus RCs are convoluted membranes and invaginated vesicle packets, which have been characterized by electron microscopy (EM).^[8^ ^]^ Flavivirus replication complexes, consisting of multiple NS proteins, assemble in RCs and catalyze viral RNA synthesis.^[^ ^9^, ^10^ ^]^ Although many efforts have been made in recent decades, the dynamic spatiotemporal organization of flavivirus RCs is still unknown, preventing us from understanding both the fundamental viral replication process and the active interaction between flavivirus and host cells. Here, we visualized the dynamic ER rearrangement by ZIKV to build viral main replication compartments (MRCs) using superresolution live‐cell imaging, revealing an efficient centralized replication mode and its underlying mechanism.

## 2 Results

### 2.1 Flavivirus Adopts Centralized Replication Mode via Concentrating Host ER to Form MRCs

**Figure 1:**
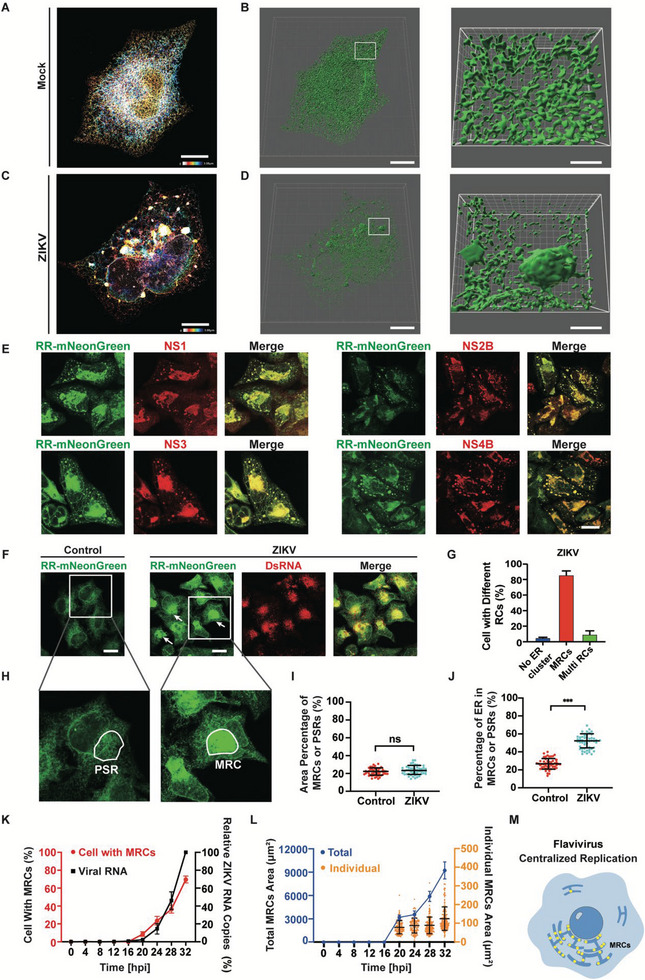
Centralized replication mode of flavivirus. A) Regular ER morphology (RR‐mNeonGreen as ER marker). RR‐mNeonGreen‐HeLa cell was imaged by STED microscope and color was coded by *z* position. Scale bar, 10 µm. B) Left, 3D rendering of the ER networks from the raw data of STED microscopy. Right, close‐up of the boxed region in the left. Scale bar, 10 µm (left) and 2 µm (right). C) ER morphology of ZIKV‐infected HeLa cell, imaged by STED microscope and color‐coded by *z* position. Scale bar, 10 µm. D) Left, 3D rendering of the ER networks from the raw data of STED microscopy. Right, close‐up of the boxed region in the left. Scale bar, 10 µm (left) and 2 µm (right). E) ZIKV NS1, NS2B, NS3 and NS4B localized at ER clusters. Scale bar, 20 µm. F) ZIKV replicated in perinuclear MRCs. The arrows indicate MRCs. Uninfected RR‐mNeonGreen cells were used as control. Scale bar, 20 µm. G) The percentage of ZIKV‐infected cells with different types of RCs. Two hundred ZIKV‐infected cells were calculated according to the type of viral RCs. H) PSRs in control cells (uninfected RR‐mNeonGreen cells) and MRCs in ZIKV‐infected RR‐mNeonGreen cells were outlined by white circle. PSRs, perinuclear similar regions. The images are the magnifications of boxed regions in (F). I) Area percentage of MRCs (ZIKV‐infected cells) or PSRs (control cells) to total cell area. J) The percentage of ER in MRCs (ZIKV‐infected cell) or PSRs (control cell). K) Time course analysis of intracellular vRNA amount (black square) and the percentage of cells containing MRCs (red dot) upon ZIKV infection. The vRNA was determined by qPCR, and vRNA amount at 32 hpi was set as 100%. L) Time course analysis of individual area (orange) and total area (blue) of MRCs. Total MRCs area was the sum of individual MRCs area of ZIKV‐infected cells from ten random fields. M) Centralized replication mode of flavivirus. Yellow dots indicate viral RCs. Data are presented as mean ± SD. The P values are obtained from two‐tailed t‐test, ^***^ *p* < 0.001. ns, not significant.

The development of various superresolution microscopy techniques has offered an opportunity to image the dynamic remodeling of the host ER by virus with high spatiotemporal resolution.^[^ ^11^, ^12^ ^]^ However, it is still challenging to monitor the abovementioned process in live cells because of the limitations on fluorescent markers to label the ultrafine and highly dynamic ER. Here, we constructed a novel ER marker, RR‐mNeonGreen, which localizes to the ER via a transmembrane region of VAMP2 and an N‐terminal arginine‐rich sequence (RR) as an ER retention signal (Figure S1A, Supporting Information).^[^ ^13^ ^]^ Intracellularly, RR‐mNeonGreen colocalized well with mCherry‐KDEL, a widely used ER marker,^[^ ^14^ ^]^ indicating that RR‐mNeonGreen perfectly labels the ER (Figure S1B–D, Supporting Information). Compared to GFP, mNeonGreen is brighter and more stable and is less sensitive to laser‐induced bleaching during long‐term imaging.^[15^ ^]^ These notable advantages make RR‐mNeonGreen competent for analyzing the dynamics of the ER ultrastructure via superresolution microscopy techniques such as stimulated emission depletion (STED) microscopy and structured illumination microscopy (SIM).^[16^ ^]^ We established a HeLa cell line stably expressing RR‐mNeonGreen to precisely visualize ER dynamics. The ER in RR‐mNeonGreen‐cells displayed regular continuous networks in the cytoplasm (**Figure** **1**A,B; Video S1, Supporting Information). Then, RR‐mNeonGreen cells were infected with ZIKV, and the morphology of the ER (represented by RR‐mNeonGreen) was visualized by STED microscope. Unlike the continuous and dense ER networks in uninfected cells, the ER networks in ZIKV‐infected cells became sparser, and multiple aggregated ER clusters appeared in the cytoplasm, many of which were located in the perinuclear region (Figure 1C). 3D rendering of the raw images showed the 3D structure of the rearranged ER networks upon ZIKV infection (Figure 1D; Video S2, Supporting Information). To confirm that these ER clusters were related to ZIKV RCs, we stained ZIKV‐infected cells with antibodies against multiple viral NS proteins (NS1, NS2B, NS3, and NS4B), which have been shown to localize at viral RCs.^[^ ^17^, ^18^ ^]^ Immunostaining confirmed that viral replication‐required NS proteins were localized in these ER clusters (Figure 1E), suggesting that these ER clusters are viral RCs.

Remarkably, RR‐mNeonGreen displayed large, bright, and dense aggregates in many ZIKV‐infected cells (Figure 1E,F), indicating that the ER was concentrated in the perinuclear region. In addition, viral dsRNA was also enriched in these ER aggregates, suggesting the presence of active viral replication (Figure 1F). We named these large perinuclear ER clusters enriched with viral NS proteins and dsRNA as viral main replication compartments (MRCs) (Figure S2A, Supporting Information), whose sizes were in the range of 50–300 µm^2^. On the other hand, there were also ER clusters with sizes <50 µm^2^, which were defined as viral RCs. Thus, three categories of ZIKV‐infected cells were divided according to viral RCs morphologies, including 1) MRC cells, cells containing MRCs with or without RCs; 2) multi‐RCs cells, cells having several RCs but no MRCs; and 3) cells having normal ER networks without ER cluster. Data showed that >80% of ZIKV ‐infected cells contained MRCs at 32 h post infection (hpi) (Figure 1G).

Given that the degree of ER clustering could be determined by the fluorescent density of RR‐mNeonGreen, thus we could quantitatively analyze the clustering of ER upon ZIKV infection. First, we calculated the percentage of MRCs area to the whole cell area (Figure 1H,I). For comparison, we used uninfected RR‐mNeonGreen cells as control and selected perinuclear similar regions (PSRs) manually (Figure 1H). The area percentages of MRCs in ZIKV‐infected cells and PSRs in control cells were similar (≈22% of the total cell area) (Figure 1I). Then we calculated the percentage of integrated RR‐mNeonGreen density in MRCs or PSRs to that in the whole cell, which indicated the degree of ER concentration in MRCs or PSRs. As shown in Figure 1J, 26.7% of cellular ER was present in PSRs in control cells. However, 50.9% of the cellular ER was concentrated in ZIKV MRCs. This suggests that ZIKV infection significantly concentrates cellular ER in viral MRCs, providing a sufficient membranous platform for flavivirus replication. To determine the relationship between viral MRCs formation and viral RNA (vRNA) synthesis, we performed time course analysis of MRCs and vRNA upon ZIKV infection. Data showed that viral MRCs formation and vRNA synthesis were highly synchronous, both rapidly rose from 16 hpi (Figure 1K). As more and more MRCs formed in ZIKV‐infected cells over time, total MRCs area (sum of MRCs area of ZIKV‐infected cells from 10 random fields) dramatically increased, accompany with the increase of individual MRCs area as well (Figure 1L). Similarly, DENV also replicated in perinuclear MRCs (Figure S2B–E, Supporting Information), suggesting that a centralized replication mode is common to flaviviruses (Figure 1M).

### 2.2 Dynamic Formation of Flavivirus MRCs

**Figure 2:**
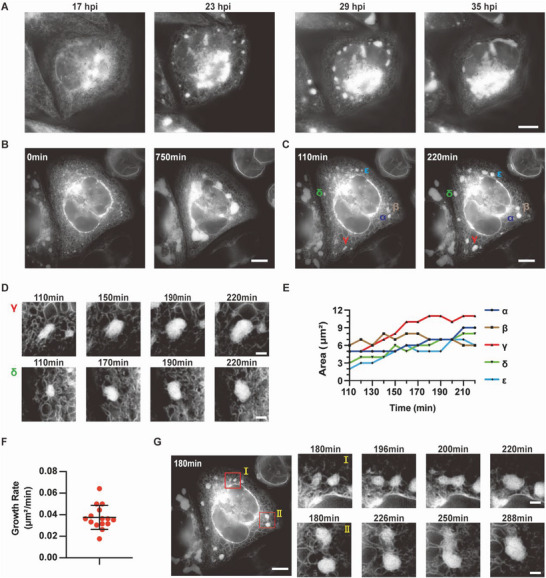
The development of ZIKV RCs (RR‐mNeonGreen as ER marker). A) Selected SIM images from time‐lapse series (2 h interval) recording ER dynamics in ZIKV‐infected HeLa cell. Scale bar, 10 µm. B) SIM images of ZIKV‐infected cell (2 min interval) at the starting point (≈16 hpi) and end point (≈28 hpi) of recording. Scale bar, 10 µm. C) SIM images (the same cell as (B)) display the status of ZIKV RCs (α‐ε are individual RCs) at indicated time points. Scale bar, 10 µm. D) The growth of selected RCs (*γ* and *δ* in (C)) during indicated periods. Scale bar, 2 µm. E) The area growth curve of indicated ZIKV RCs (α‐ε in (C)). F) Area growth rate of ZIKV RCs. G) Selected SIM images from time‐lapse series recording the merging of ZIKV RCs (the same cell as (B)). Scale bar, 10 µm (left) and 2 µm (right).

Next, to visualize the dynamics of flavivirus RCs, we performed time‐lapse imaging by SIM. We monitored the long‐term ER dynamics (RR‐mNeonGreen) of ZIKV‐infected cells from 16 hpi with 2 h intervals, according to the time course study of MRCs formation. Several ZIKV RCs were originated at multiple sites within 20 hpi; these RCs included a few large perinuclear RCs and multiple small RCs at the peripheral ER. In the following hours, most RCs disappeared, and the central MRCs were established (**Figure** **2**A). The ultrastructure of ZIKV RCs was also examined by EM, which validated that the perinuclear MRCs contained characteristic structures, i.e., invaginated vesicle packets (VPs) and convoluted membranes (CMs) (Figure S3, Supporting Information). The perinuclear MRCs might represent a mature stage of ZIKV RCs, considering the development of RCs over time (Figure 2A). Based on the above observation, we proposed a model in which ZIKV RCs originate from multiple ER clusters at different sites and gradually converge into perinuclear MRCs.

To verify our hypothetical model, ER dynamics in ZIKV‐infected cells was visualized by SIM at a shorter time interval of 2 min. We tracked the dynamics from the early stage (≈16 hpi), when only tiny RCs appeared sporadically, to the late stage, when large perinuclear MRCs formed (≈28 hpi, Figure 2B; Video S3, Supporting Information). As the ER is a highly dynamic organelle, ZIKV RCs derived from the ER inherit the characteristics. We measured the size of ZIKV RCs over time and found that some RCs actively increased in size (Figure 2C–E; Videos S4–S6, Supporting Information). The average area growth rate of these RCs was 0.0375 µm^2^ min^−1^ (Figure 2F). Additionally, ZIKV RCs moved around and frequently merged with each other to form larger RCs, providing an alternative way to quickly expand RCs (Figure 2G; Videos S7 and S8, Supporting Information). However, division event of RCs has been rarely recorded.

**Figure 3:**
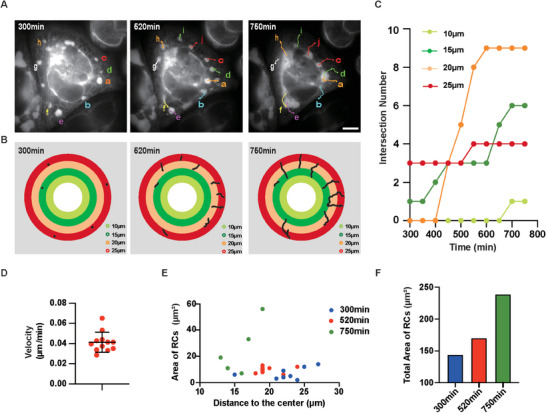
Directional movements of ZIKV RCs toward nucleus with the increasing of RCs areas. A) RCs movements were traced by ImageJ. Scale bar, 10 µm. B) Trajectories of RCs in (A) were analyzed and intersections with rings were recorded. Color is coded by radius. C) The intersections of RCs trajectories with each ring were counted at indicated time point and plotted over time. The colors of dotted lines are in accord with colors of rings in (B). D) Motion velocity of ZIKV RCs. RCs from five cells were analyzed. E) The distance to nuclear center and area of individual RCs were measured and plotted. Each dot indicates one RCs. F) The area of total RCs increased over time.

As ZIKV RCs originated from both perinuclear and peripheral ER, the movements of RCs could be important for the centralization. We analyzed the trajectory of ZIKV RCs (**Figure** **3**A). For simplicity, we used rings with different colors to indicate the distances to cell center (Figure 3B). Increasing intersection numbers of RCs trajectories with rings over time demonstrated that ZIKV RCs moved toward the nucleus and finally converged in the perinuclear region (Figure 3B,C). The motion velocity of ZIKV RCs was 0.0413 µm min^−1^ (Figure 3D). We also quantified the RCs number and the area of individual and total RCs in the same ZIKV‐infected cell over time. While ZIKV RCs moved toward the nucleus, the number of RCs decreased due to merging, but the area of individual RCs and total RCs increased (Figure 3E,F). These data comprehensively delineate the overall process of ZIKV MRCs formation, including their origination from multiple ER clusters, their expansion via individual growth and mutual merging, and their convergence into perinuclear MRCs. Importantly, recording the formation of ZIKV perinuclear MRCs by live‐cell imaging identifies a dynamic mechanism for the centralized replication of flavivirus.

### 2.3 Dynamic Motions of Viral RCs are Driven by NS Proteins

**Figure 4:**
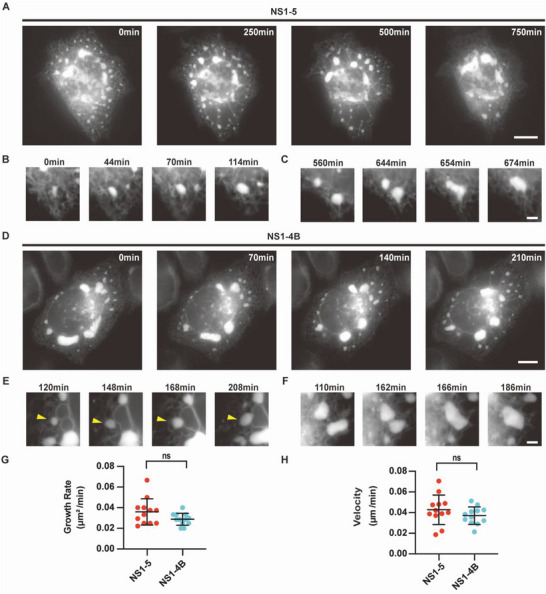
Dynamics of ER clusters is driven by ZIKV NS proteins (RR‐mNeonGreen as ER marker). A) Selected SIM images from time‐lapse series recording the ER dynamics in cell co‐expressing ZIKV NS1‐5 and RR‐mNeonGreen. Scale bar, 10 µm. B) The growth of ER cluster. C) The merging of ER clusters. Scale bar, 2 µm. D) Selected SIM images from time‐lapse series recording the ER dynamics in cell co‐expressing ZIKV NS1‐4B and RR‐mNeonGreen. Scale bar, 10 µm. E) The growth of ER cluster. F) The merging of ER clusters. Scale bar, 2 µm. G) Area growth rate of ER clusters induced by ZIKV NS proteins. H) Motion velocity of ER clusters induced by ZIKV NS proteins. ns, not significant.

Given that flavivirus NS proteins are responsible for viral replication and that certain NS proteins specifically play crucial roles in the biogenesis of viral replication compartments,^[^ ^18^, ^19^, ^20^ ^]^ we examined whether the ZIKV NS1‐5 proteins dynamically remodel the host ER as ZIKV does. We cotransfected HeLa cells with plasmids encoding ZIKV NS1‐5 and RR‐mNeonGreen and found that NS1‐5 expression induced the formation of multiple ER clusters (**Figure** **4**A). Immunostaining demonstrated that ZIKV NS proteins localized at these ER clusters (Figure S4A,B, Supporting Information). In addition, EM images implied that NS1‐5 could induce the formation of CMs‐ and VPs‐ like structures resembling ZIKV RCs (Figure S4D, Supporting Information).^[21^ ^]^ The ER clusters induced by ZIKV NS1‐5 proteins also grew and merged (Figure 4B,C). Directional movement toward the nucleus and merging of ER clusters resulted in perinuclear convergence. These data suggest that ZIKV NS1‐5 can dynamically remodel the host ER to form viral RCs‐like structures. As a viral RNA polymerase, NS5 localizes to the cytosol and nucleus and does not exhibit membrane rearrangement capability.^[^ ^22^, ^23^ ^]^ Moreover, evidence showed that the biogenesis of ZIKV RCs is not affected by deficiency of NS5 activity.^[21^ ^]^ Then we expressed ZIKV NS1‐4B and RR‐mNeonGreen in HeLa cells and monitored ER dynamics. NS1‐4B also induced ER clustering, merging and perinuclear convergence (Figure 4D–F; Figure S4A,C,E, Supporting Information). Apparently, NS5 may not be directly involved in the biogenesis of ZIKV RCs or at least not affect the related dynamics, but a positive role of NS5 in facilitating viral RCs formation cannot be ruled out. The structural and motional features of the ER clusters induced by ZIKV NS proteins are very similar to those of ZIKV RCs (Figure 4G,H; Figure S5B,C, Supporting Information), suggesting that ZIKV NS proteins are sufficient to induce the formation of viral MRCs.

### 2.4 ZIKV RCs Movement is Independent of Microtubules and Actin

**Figure 5:**
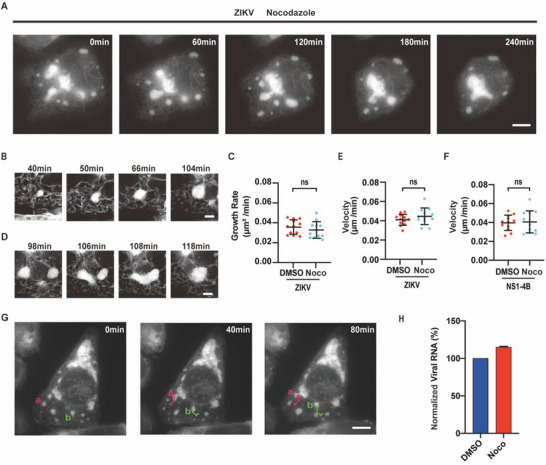
The movement of ZIKV RCs is independent of microtubules (RR‐mNeonGreen as ER marker). A) Selected SIM images from time‐lapse series recording ER dynamics in ZIKV‐infected cell after treatment of nocodazole. ZIKV infected cells were treated with nocodazole (1 µg mL^−1^) at 16 hpi for 30 min. Scale bar, 10 µm. B) The growth of ZIKV RCs in the presence of nocodazole. Scale bar, 2 µm. C) Area growth rate of ZIKV RCs in the presence of nocodazole. D) The merging of ZIKV RCs in the presence of nocodazole. Scale bar, 2 µm. E) Motion velocity of ZIKV RCs in the presence of nocodazole. F) Motion velocity of ZIKV NS1‐4B‐induced ER clusters upon the treatment of nocodazole. G) Trajectories of ZIKV RCs upon the treatment of nocodazole. Scale bar, 10 µm. H) Nocodazole did not inhibit ZIKV replication after viral entry. Total RNA was extracted from DMSO or nocodazole treated ZIKV‐infected cells and the amount of viral RNA was determined by RT‐qPCR.

Previous studies have shown that ER distribution and movement might be influenced by microtubules but not by actin in mammalian cells.^[^ ^24^, ^25^ ^]^ We sought to determine whether the dynamic activities of ZIKV RCs depend on microtubules. Nocodazole, an inhibitor of microtubules polymerization, was used to treat ZIKV‐infected cells and the motion of ZIKV RCs was tracked.^[^ ^26^, ^27^ ^]^ The working concentration of nocodazole was determined according to the morphology of RR‐mNeonGreen stable cell after treatment (Figure S5A, Supporting Information). As cells gradually turned round upon nocodazole treatment, live‐cell imaging could be performed for only 2–6 h. The overall dynamic features of ZIKV RCs in the presence of nocodazole were similar to those in the absence of nocodazole (**Figure** **5**A). In detail, growth and merging of ZIKV RCs were observed upon nocodazole treatment (Figure 5B,D). Moreover, nocodazole did not change the area growth rate or motion velocity of ZIKV RCs (Figure 5C,E; Figure S5B,C, Supporting Information). To examine whether the movements of ER clusters induced by NS1‐4B depend on microtubules, HeLa cells expressing NS1‐4B and RR‐mNeonGreen were treated with nocodazole. Data showed that the velocity of ZIKV NS1‐4B‐induced ER clusters was not affected by nocodazole (Figure 5F). Meanwhile, the directional movement toward nucleus of ZIKV RCs remained unchanged in the presence of nocodazole (Figure 5G). Quantification of viral RNA further confirmed that ZIKV replication was insensitive to nocodazole after viral entry (Figure 5H). In addition to nocodazole, Lexibulin (an irreversible microtubule polymerization inhibitor) and Paclitaxel (a microtubule stabilizer) did not affect ZIKV RCs movements (Figure S5D, Supporting Information). Collectively, these results suggest that the dynamics of ZIKV RCs are independent of microtubules. Similarly, Latrunculin A (an actin polymerization inhibitor) was tested, and it had no effect on the motion velocity of ZIKV RCs (Figure S5D, Supporting Information). The lack of dependence on microtubules and actin suggests that the dynamic activities of ZIKV RCs are more related to the ER membrane tension and other factors such as lipid synthesis and ER structural proteins. Considering that a couple of ZIKV NS proteins have been proven to be capable of altering the ER membrane structure,^[^ ^18^, ^19^, ^20^ ^]^ it is rational to hypothesize that this kind of membrane tension might arise from the interaction between ZIKV NS proteins and the ER membrane.

### 2.5 Disrupting MRCs Formation Inhibits Viral Replication

**Figure 6:**
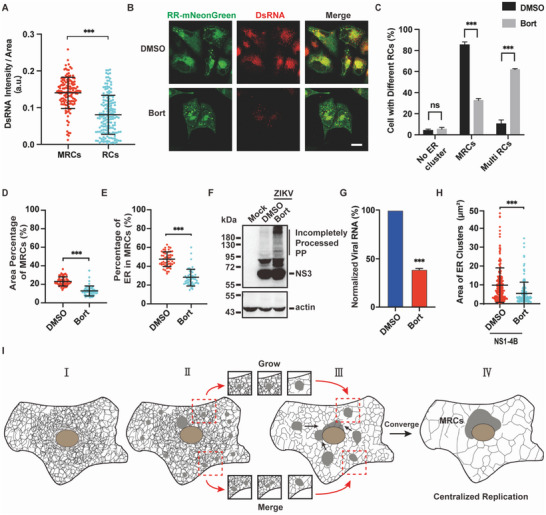
Centralized replication mode favors flavivirus replication. A) Analysis of dsRNA intensity per unit area in MRCs and RCs. DsRNA intensity per unit area was calculated by dividing the integrated intensity of dsRNA in MRCs or RCs by the area of MRCs or RCs. B) RCs in ZIKV‐infected cells after treatment with DMSO or bortezomib (Bort, 20 nm). Scale bar, 20 µm. C) The percentage of ZIKV‐infected cells with different types of RCs after treatment with DMSO or bortezomib (20 nm). Two hundred ZIKV‐infected cells were calculated according to the type of viral RCs. D,E) Area percentage of MRCs to the total cellular area D) and the percentage of ER in MRCs E) upon treatment with DMSO or bortezomib. F) Bortezomib (20 nm) impaired ZIKV polyproteins processing. PP, polyproteins. G) Bortezomib (20 nm) inhibited ZIKV replication. H) The area of ER clusters induced by NS1‐4B upon bortezomib treatment. I) Biogenesis of flavivirus RCs and formation of MRCs (I–IV): Upon flavivirus infection, the host ER (I) start to form multiple clusters, i.e., viral RCs (II). Then viral RCs expand via growth and merging (III). Meanwhile, ZIKV RCs directionally move toward the nucleus and converge into perinuclear MRCs in the end (IV). Data are presented as mean ± SD. The P values are obtained from two‐tailed t‐test, ^***^ *p* < 0.001. ns, not significant.

The synchronization of vRNA synthesis and MRCs formation implies MRCs as efficient viral replication platform. To prove this, we measured dsRNA intensity per unit area in MRCs or RCs respectively. The intensity of dsRNA per unit area in MRCs was stronger than that in RCs, indicating more efficient viral replication in MRCs (**Figure** **6**A). Our recent study showed that bortezomib, a proteasome inhibitor, can induce ubiquitination and aggregation of ZIKV NS3 protease, thus interfering with the processing of flavivirus polyproteins (PP) into individual NS proteins.^[28^ ^]^ Therefore, we wonder whether the disturbance in NS protein processing caused by bortezomib also affects the formation of ZIKV MRCs. Bortezomib (20 nm) treatment resulted in more distributed individual viral RCs in ZIKV‐infected cells, effectively hindering the formation of central MRCs (Figure 6B,C). Furthermore, compared to DMSO, bortezomib significantly reduced both the area percentage of MRCs and the percentage of ER in MRCs (Figure 6D,E). Western blotting confirmed that bortezomib impaired ZIKV PP processing, leading to the accumulation of incompletely processed viral PP (Figure 6F). As a result, ZIKV replication was inhibited (Figure 6G; Figure S6A, Supporting Information). Likewise, bortezomib treatment also decreased the size of NS1‐4B‐induced ER clusters (Figure 6H).

Lastly, we wonder whether blocking lipid synthesis could affect viral MRCs formation. TVB‐2640, an inhibitor of fatty acid synthase (FASN), was added to ZIKV‐infected cells at 8 hpi. Data showed that TVB‐2640 decreased the percentage of cells containing MRCs and the area of RCs, thus inhibiting vRNA synthesis (Figure S6B–E, Supporting Information). Therefore, blocking cellular lipid synthesis also impaired ZIKV replication via disrupting MRCs formation.

Altogether, these results suggest that centralized replication of flavivirus in MRCs is an efficient replication mode and that disrupting the formation of flavivirus MRCs could be a promising antiviral strategy.

## 3 Discussion

Positive‐strand RNA viruses usually replicate in membrane surrounded RCs, which are derived from host organelles such as ER, Golgi, mitochondria, etc.^[3^ ^]^ Multiple positive‐strand RNA viruses remodel ER to form two types of structurally distinguishable RCs, invaginated vesicles of flavivirus and double‐membrane vesicles (DMVs) of coronavirus and HCV.^[^ ^4^, ^29^, ^30^ ^]^ Here, using superresolution live‐cell imaging we visualized dynamic ER remodeling process by flavivirus, implying a centralized replication modes of flavivirus at the ER. Specifically, flavivirus centralizes host ER in perinuclear viral MRCs, which are formed via the growth, merging, directional movement, and convergence of individual RCs (Figure 6I). Previous studies reported that flavivirus RCs came from rough and smooth ER,^[^ ^8^, ^31^ ^]^ we found that flavivirus RCs could originate indiscriminately from perinuclear ER (most is rough ER) as well as peripheral ER (most is smooth ER), and these RCs eventually converge together. In this way, flavivirus hijacks and concentrates ≈50% of intracellular ER in perinuclear region, thus providing sufficient membrane source for viral RCs and making the most efficient use of host protein synthesis machinery. Flavivirus centralized MRCs are efficient platforms for viral RNA synthesis at which viral replication‐required NS proteins, genomic RNA and substrates could be highly concentrated and actively exchanged, so that viral replication efficiency can be increased.

Our data also suggests that viral NS proteins are primary driving factors contributing to dynamic motions of flavivirus RCs. Flavivirus NS1‐5 proteins induced ER clustering, and the dynamics of ER clusters induced by NS proteins is similar to that of flavivirus RCs. The division of ER cluster was hardly observed, implying the irreversible trend of ER centralization induced by flavivirus NS proteins. Among flavivirus NS proteins, NS1 is a membrane‐binding protein that localizes in ER lumen, whose expression induced robust ER clustering.^[18^ ^]^ NS4A and 4B were also reported to participate in ER rearrangement.^[^ ^19^, ^20^ ^]^ It is reasonable to assume that the formation of viral RCs depends on the cooperation of these NS proteins. To fully understand viral RCs biogenesis and dynamics, the exact role of each NS protein needs to be identified.

In mammalian cells, ER could move along microtubules,^[^ ^25^, ^27^ ^]^ but we found that dynamic activities of flavivirus RCs were not instantaneously affected by microtubules or actin inhibitors, suggesting that the motions of RCs are independent of microtubules and actin. Due to cell morphological changes, our live‐cell imaging can be performed for only a few hours after the treatment of microtubules or actin inhibitors. So, it is not clear whether the disruption of microtubules or actin dynamics would affect viral RCs movement in a longer time.

As the efficient replication platform, MRCs are crucial for flavivirus replication and could be a potential anti‐flavivirus target. In the present study, we found that two small molecule compounds disrupted viral MRCs formation. Previous studies of our group and other groups have identified bortezomib as a broad inhibitor against flaviviruses including ZIKV, DENV and JEV in cells as well as in animal models.^[^ ^32^, ^33^, ^34^, ^35^, ^36^ ^]^ We found that bortezomib interfered with flavivirus polyprotein processing, hindered the formation of flavivirus MRCs and inhibited viral replication at low concentration. In addition, blocking cellular lipid synthesis by FASN inhibitor TVB‐2640 also affected viral MRCs formation and inhibited viral replication. A recent study showed that FASN inhibitors blocked SARS‐CoV‐2 replication, although the mechanism remained unclear.^[37^ ^]^ As SARS‐CoV‐2 replicates in DMVs, it is possible that FASN inhibitors may disturb SARS‐CoV‐2 RCs formation. Therefore, disruption of viral RCs formation could be an important inhibitory mechanism and a broad antiviral strategy targeting viral replication in membranous compartments.

## 4 Conclusion

In summary, our study provides unprecedented information about the dynamic organization of flavivirus RCs by superresolution live‐cell imaging, which hasn't been revealed by EM or other imaging techniques based on fixed cells. Furthermore, we unravel the dominant roles of NS proteins in the formation of flaviviral MRCs. These findings shed light on the active interaction between flavivirus and host ER and facilitate the development of new antiviral strategies.

## Conflict of Interest

The authors declare no conflict of interest.

## Author Contributions

Y.C. and K.H. contributed equally to this work. L.S. and Y.C. conceived the project and designed the experiments. Y.C., K.H., J.K., S.H., and Y.Y. performed experiments. C.F.Q. contributed to experiments and data analysis. Y.C. and L.S. wrote the manuscript and all other authors edited the manuscript.

## Supporting information

Supporting Information

Supplemental Video 1

Supplemental Video 2

Supplemental Video 3

Supplemental Video 4

Supplemental Video 5

Supplemental Video 6

Supplemental Video 7

Supplemental Video 8

## Data Availability

The data that support the findings of this study are available in the supplementary material of this article.
