# Supplementary material for: Flavivirus Concentrates Host ER in Main Replication Compartments to Facilitate Replication
Source: Adv Sci (Weinh). 2023 Oct 27;10(36):2305093. doi: 10.1002/advs.202305093 (PMC10754076; doi:10.1002/advs.202305093)
Supplement: Supplementary file 1 — Supporting Information [file ADVS-10-2305093-s009.pdf]

ADVANCED SCIENCE
Open Access

Supporting Information

for *Adv. Sci.*, DOI 10.1002/advs.202305093

Flavivirus Concentrates Host ER in Main Replication Compartments to Facilitate Replication

Yali Ci, Kai Han, Jie Kong, Shuhan Huang, Yang Yang, Cheng-Feng Qin and Lei Shi**

Materials and Methods

Cell culture

Human HeLa (ATCC CCL-2) cells and Huh-7 cells were grown at 37 ℃ with 5 % CO_2 in DMEM supplied with 10% FBS.

Virus strains

The Zika virus strain (FSS13025) and the DENV-2 virus strain (DENV2_China_SZ_2015) were acquired from the State Key Laboratory of Pathogen and Biosecurity in Beijing, China.

Antibodies

Antibodies used in this study were as follows: ZIKV NS1 (BioFront, BF-1225-06 and BF-1225-36), ZIKV NS3 (GeneTex, GTX133309), ZIKV NS2B (GeneTex, GTX133308), ZIKV NS4B (GeneTex, GTX133311), ZIKV NS5 (BioFront, BF-8B8), β-actin (Sigma-Aldrich, A5441), Calreticulin (Abcam, ab92516 and ab2907), Cy3-conjugated goat anti-rabbit (Jackson ImmunoResearch), Alexa Fluor 594-conjugated Goat anti-mouse (Invitrogen), and Alexa Fluor 647-conjugated Goat anti-mouse (Invitrogen).

Reagents

ZIKV-infected cells were treated with bortezomib (20 nM, Selleck, S1013) or TVB-2640 (1μM, Selleck, S9714) at 8 hpi and cells were harvested or fixed at 24 or 32 hpi. Upon treatment with microtubules or actin inhibitors, cells gradually turned round over the time in glass-bottom chamber, impeding the long-term live-cell imaging. To perform the imaging, cells were treated with Nocodazole (Selleck, S2775), Paclitaxel (Selleck, S1150), Lexibulin (MCE, HY-10498), Latrunculin A (Abcam, ab144290) for 30 min at 16-20 hpi. Then the drug was removed and cells were imaged for 2-6 hours.

RR-mNeonGreen plasmid construction

The coding sequence of fluorescence protein mNeonGreen was inserted into pcDNA3.1(+) vector. Then DNA coding N-terminal ER retention signal (MHRRRSRSCREDQKPV) with Kozak translation initial sequence was inserted to the upstream of mNeonGreen sequence. And coding sequence of VAMP2 TM region (KYWWKNLKMMIILGVICAIILIIIIVYFST) was introduced to the downstream of mNeonGreen sequence to drive mNeonGreen targeting ER membrane.

Immunofluorescence

HeLa or Huh-7 cells infected by ZIKV/DENV or transfected by NS protein were fixed with 3% PFA containing 0.1% glutaraldehyde at RT for 10 min. After three times of washing by PBS, cells were permeabilized by 0.2% Triton-X100 diluted in PBS at RT for 10 min, and blocked by 3% BSA for 30 min. Then cells were incubated with indicated primary antibodies at 37 °C for 2 h followed by secondary antibodies at 37 °C for 1 h. Finally, cells were mounted by ProLong Diamond mountant (Invitrogen).

EM

HeLa cells were seeded in 35-mm dishes and fixed with 2.5 % glutaraldehyde in PBS followed by post-fixation with 1% osmium containing 1.5% potassium ferrocyanide. Then cells were dehydrated with gradient ethanol (50 %, 70 %, 90 %, 95 %, 100 %). Cells were infiltrated with and embedded in SPON812 resin in situ. After polymerization, embedded monolayer cells were cut to 70nm-thick ultrathin sections by diamond knives. The indicated sections were laid on copper grids and double stained with uranyl acetate and lead citrate. EM sample preparation was performed at the Center of Biomedical Analysis, Tsinghua University. And EM images were captured by electron microscope H-7650.

Live-cell imaging by Structured illumination microscopy (SIM)

HeLa cells were seeded into confocal imaging chamber and infected with ZIKV or

co-transfected with RR-mNeonGreen (50 ng) and ZIKV NS protein expressing plasmids (500 ng). Live-cell imaging was started from about 16 hpi (hours post infection) /hpt (hours post transfection). Time-lapse imaging was done on DeltaVision OMX imaging system (GE Healthcare) with the parameters: NA = 1.42 (oil immersed), Ex = 488nm, single pixel size of the detector =0.039μm, 2-hour or 2-min interval for 2D-SIM mode. Cells were imaged in phenol red-free DMEM and incubated at 37°C and 5% CO_2.

SIM images were reconstructed by SoftWoRx 7.2.2 (GE Healthcare) with main parameters: Wiener Filter Constant = 0.01, Bias Offset =35, Use Channel-Specific OTF and K0 Angles. K0 Angles = -0.810000, -1.855000, -2.890000.

When capturing images of long-term live cells, we chose the largest cross-section of the nuclei as the focal plane and utilized the auto-focusing strategy of DeltaVisionOMX-UltimateFocus. The focusing parameters are set as follows: the move threshold is 100 nm, the maximum iteration is 1, and auto-focusing is conducted every 6 time points.

For nocodazole experiment, ZIKV-infected cells were treated with nocodazole (1μg/ml) for 30min at 16 hpi, and then fresh phenol red-free DMEM was replaced and cell was imaged by SIM.

MRCs (PSR) analysis

The regions of MRCs and whole cell were measured by drawing a freehand ROI around the MRCs and cells using ImageJ. For control cells (uninfected cells), which had no MRCs, a similar ROI in perinuclear region was drawn and defined as perinuclear similar region (PSR). The area percentage of MRCs (PSR) was calculated as the area of MRCs (PSR) divided by that of whole cell. The percentage of ER in MRCs (PSR) was calculated as the integrated density of ER fluorescence (RR-mNeonGreen or ER marker) in MRCs (PSR) regions divided by the that of whole cell (ImageJ). MRCs or PSR from 50 cells were analyzed.

3D-STED imaging

3D-STED images were acquired by Leica TCS-SP8 STED 3X with HC PL APO CS2 100x/1.4 oil objective. Serial Z stack sectioning was done with 488nm excitation laser and 592nm STED laser at 140 nm intervals and imaging settings were as follows: xy pixel size 28nm, pinhole 1AU, speed 400Hz. Z-stack images were processed by Lightning module.

Imaging data processing

2D-SIM images were deconvoluted by Sparse deconvolution software (developed by Liangyi Chen lab, Institute of Molecular Medicine, Peking-Tsinghua Center for Life Sciences) and time-lapse images were subjected to CorrectBleach plugin in ImageJ (NIH). For 3D images, the color-coding by depth is projected by ImageJ and 3D construction was processed by Imaris x64 9.7.2 software.

ER cluster tracking

RCs or ER clusters trajectories were generated by Manual Tracking plugin in ImageJ. Tracking was performed on time-lapse images at 10 min per frame with lifetime at least 60 min. The moving velocity of RCs or ER clusters from 5-12 cells was calculated.

ER clusters directional movement analysis

ER clusters trajectories were made by Manual Tracking as described above and displayed as Lines. The trajectories image was analyzed by Sholl Analysis plugin in ImageJ. Manually mark the center of nuclei and set the ending radius as 700 pixels. Record the number of trajectories intersecting with circles with different radii.

ER cluster area growth analysis

ER cluster/RCs area was measured by drawing a freehand ROI around the cluster and then record the area of ROI in ImageJ. The growth rate was calculated as (end area-start area)/time. ER clusters from 5-12 cells were recorded and analyzed.

Viral RNA quantification

HeLa cells were infected with ZIKV. Total RNA was extracted by RNAfast200 kit (FASTAGEN). RT-qPCR was performed by FastKing one-step RT-qPCR kit (TIANGEN).

Viral dsRNA quantification in MRCs or RCs

ER labeled by RR-mNeonGreen was segmented by Fiji. For MRCs region identification, ER channel was filtered by Gaussian Blur with Sigma (Radius) 5.00. Then make binary by manually set threshold and select MRC region manually. For RCs identification, ER channel was filtered by Gaussian Blur with Sigma (Radius) 2.00. Then make binary by manually set threshold and select RCs region manually. The integrated signal intensities of dsRNA in MRCs or RCs regions are calculated by Fiji and the dsRNA intensity per unit area was acquired by dividing integreated dsRNA intensity in each MRCs or RCs by the area.

Supplemental information

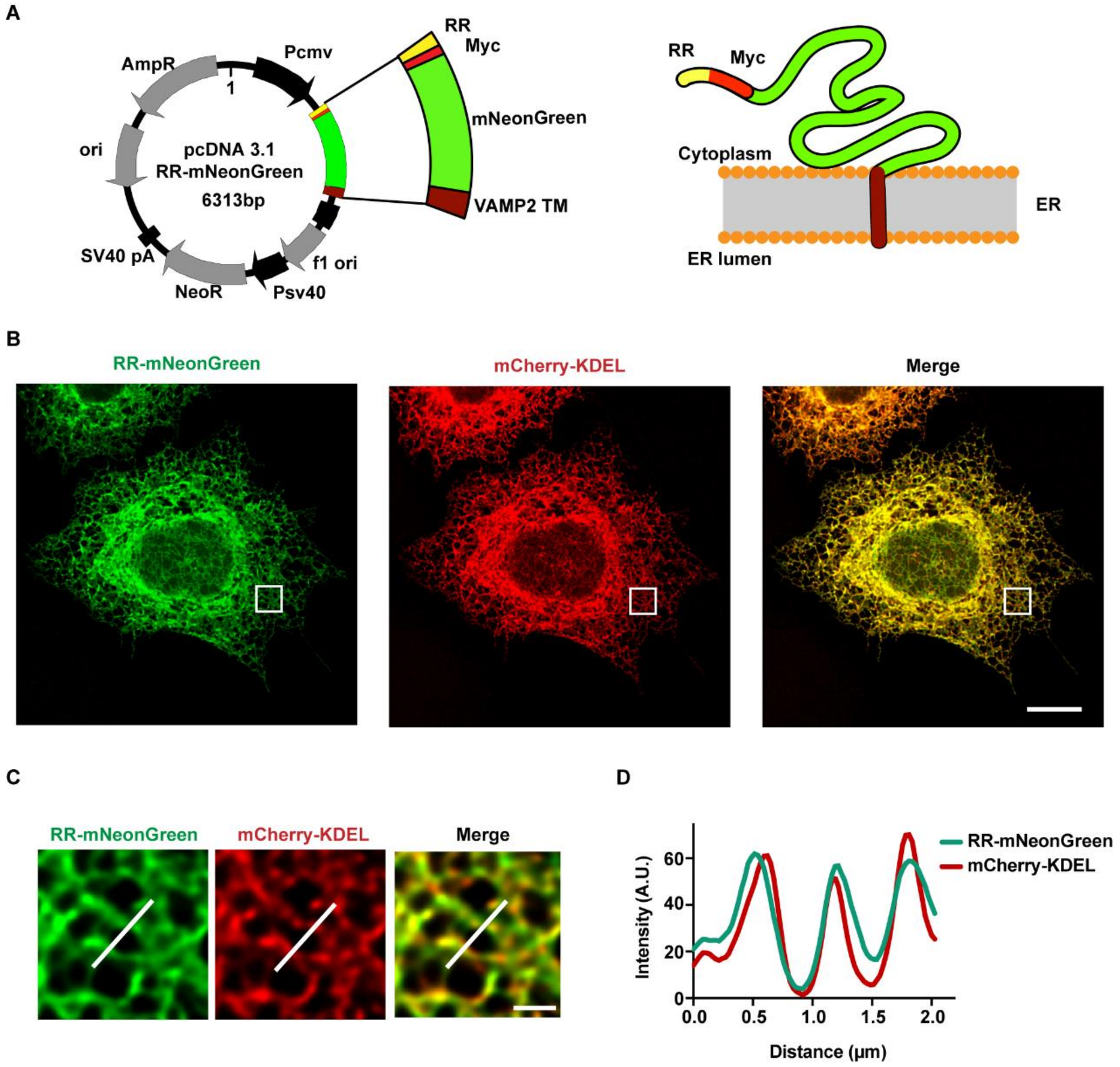

Figure S1 RR-mNeonGreen is a novel, reliable ER marker.

(A) Left, the map of plasmid encoding RR-mNeonGreen. RR-mNeonGreen has a N-terminal arginine-rich (RR) region as ER retention signal, followed by a Myc-tag, mNeonGreen fluorescent protein and a transmembrane (TM) region from VAMP2 at the C-terminus. Right, topology of RR-mNeonGreen on ER membrane.

(B) Localization of RR-mNeonGreen and mCherry-KDEL in HeLa cells. Scale bar, 10 μm. (C and D) Co-localization analysis of RR-mNeonGreen and mCherry-KDEL.

(C) A close-up image of the boxed region in (B). Scale bar, 1 μm.

(D) Analysis of green and red fluorescent signal along the line drawing in (C).

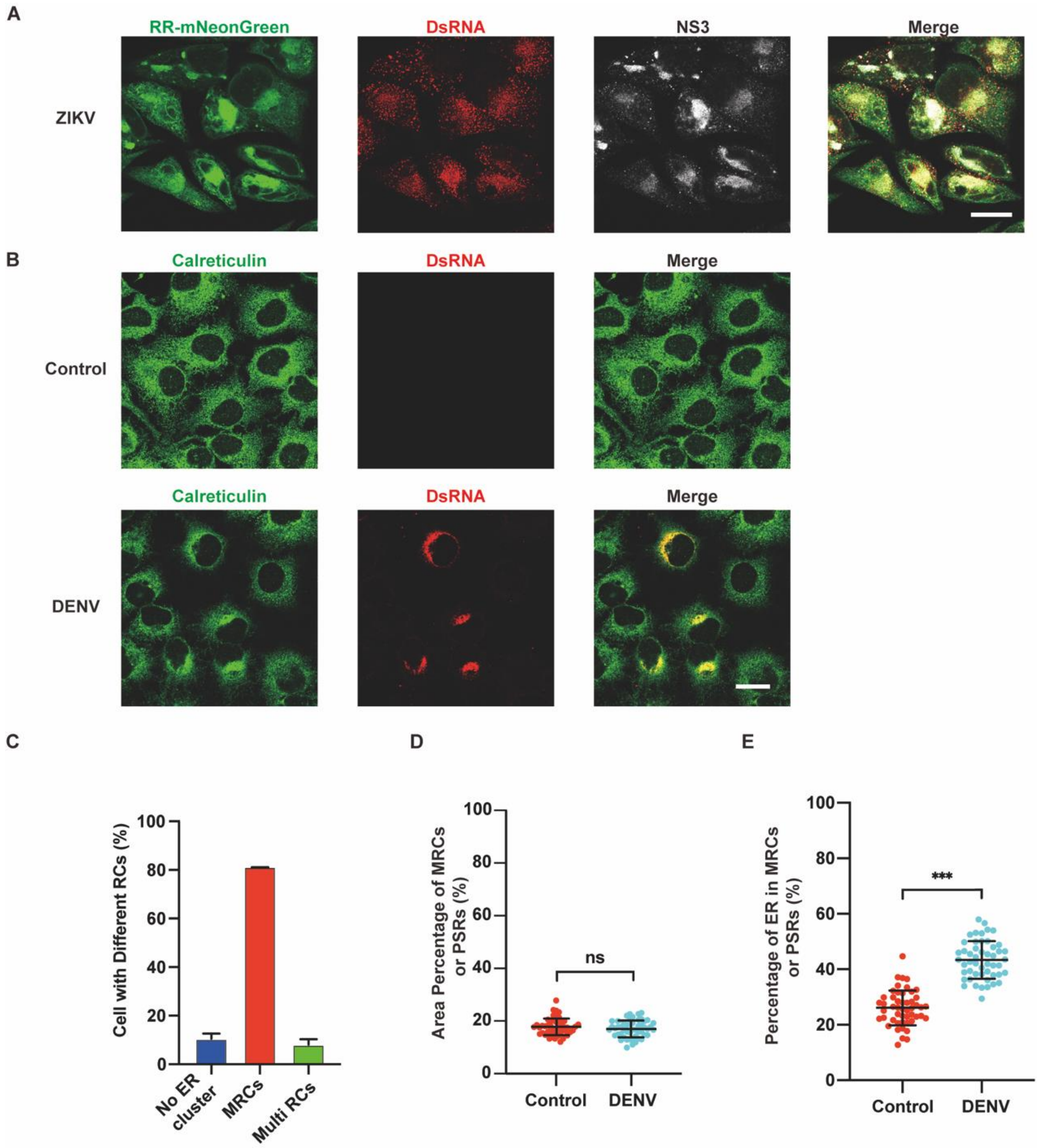

Figure S2 ZIKV and DENV replicated in perinuclear MRCs.

(A) ZIKV NS3 and DsRNA were colocalized at MRCs. Scale bar, 20 μm.

(B) Viral RCs in DENV-infected cells. Uninfected (control) or DENV-infected Huh-7 cells were stained with calreticulin (ER marker) and dsRNA antibodies. Scale bar, 20 μm.

(C) The percentage of DENV-infected cells with different types of RCs. Two hundred DENV-infected cells were calculated according to the type of viral RCs.

(D and E) The area percentage of MRCs (DENV-infected cells) or PSRs (control cells) to total cell area (D) and the percentage of ER in MRCs (DENV-infected cells) or PSRs (control cells) (E).

Data are presented as mean ± SD. The P values are obtained from two-tailed t-test, ***$P < 0.001$. ns, not significant.

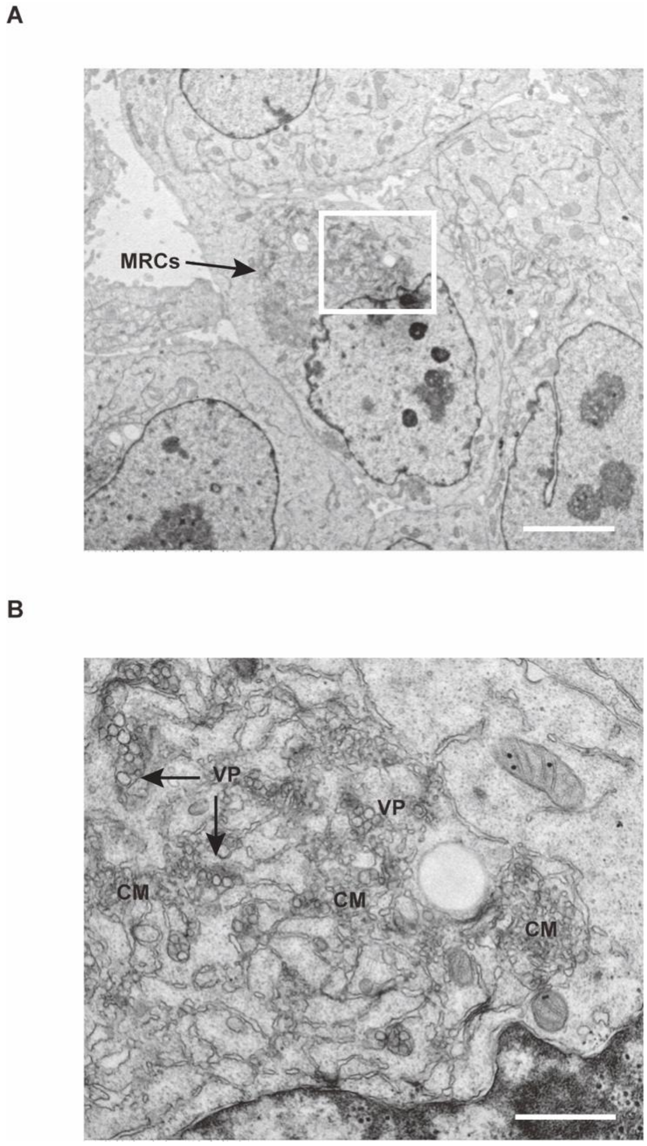

Figure S3 EM demonstrated the ultrastructure of ZIKV perinuclear MRCs.

(A) ZIKV induced MRCs in perinuclear region. Scale bar, 5 μm.

(B) Magnification of boxed region in (A). Scale bar, 1 μm.

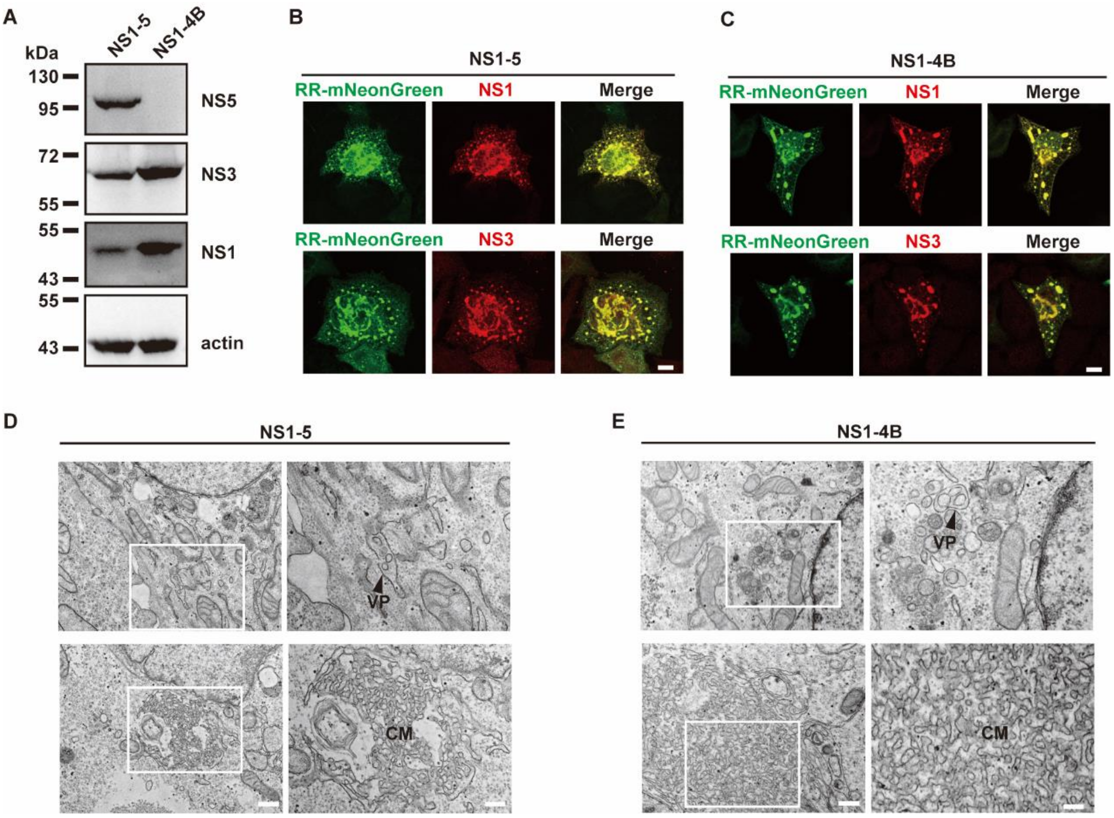

Figure S4 Nonstructural proteins of ZIKV induce RCs-like structures.

(A) Western blotting to detect the expression of indicated ZIKV NS proteins in HeLa cells transfected with plasmids encoding ZIKV NS1-5 or NS1-4B.

(B and C) ZIKV NS1 and NS3 localized at ER clusters in HeLa cells expressing NS1-5 (B) or NS1-4B (C). Scale bar, 10 µm.

(D and E) EM images showed that ZIKV NS1-5 (D) and NS1-4B (E) induced VP and CM resembling viral RCs. Right panels, close-up of boxed regions in the left images. Scale bar, 500 nm (left), 250 nm (right).

A

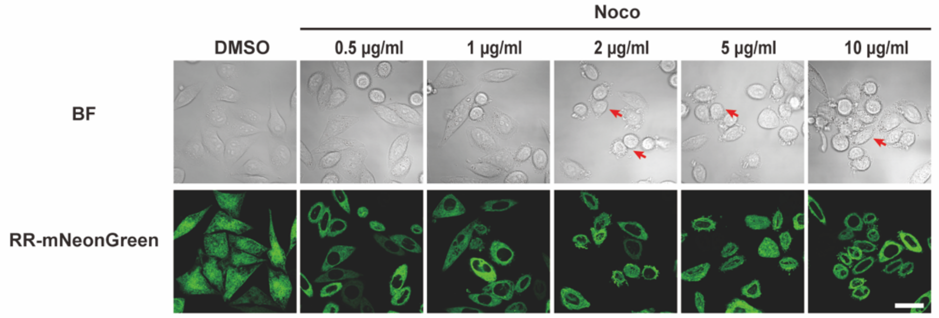

B

	ZIKV	ZIKV-Noco	NS1-5	NS1-4B
Growth Rate (μm²/min)	0.0375 ± 0.0116	0.0329 ± 0.0083	0.0360 ± 0.0127	0.0288 ± 0.0058
P		0.2472	0.7404	0.0220
Significance		n.s.	n.s.	*

C

	ZIKV	ZIKV-Noco	NS1-5	NS1-4B
Velocity (μm/min)	0.0413 ± 0.0098	0.0446 ± 0.0086	0.0428 ± 0.0143	0.0371 ± 0.0084
P		0.3882	0.7677	0.2685
Significance		n.s.	n.s.	n.s.

D

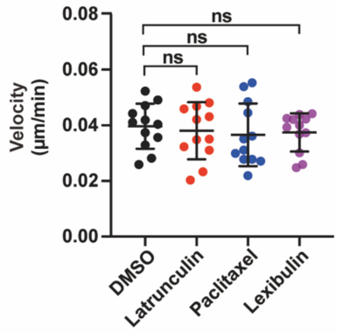

Figure S5 RCs movement is independent of microtubules and actin.

(A) Response of RR-mNeonGreen stable cells to different concentration of nocodazole. Bright-field and RR-mNeonGreen fluorescent images were taken at 2 hours after the treatment of nocodazole. BF, bright-field. Scale bar, 50 μm.

(B) Comparison of area growth rate of ZIKV RCs (with or without nocodazole) and ER clusters induced by NS1-5 or NS1-4B.

(C) Comparison of motion velocity of ZIKV RCs (with or without nocodazole) and

ER clusters induced by NS1-5 or NS1-4B.

(D) Motion velocity of ZIKV RCs upon the treatment of DMSO, Latrunculin A (0.2 μg/ml), Paclitaxel (12.5 μM), and Lexibulin (100 nM). ns, not significant.

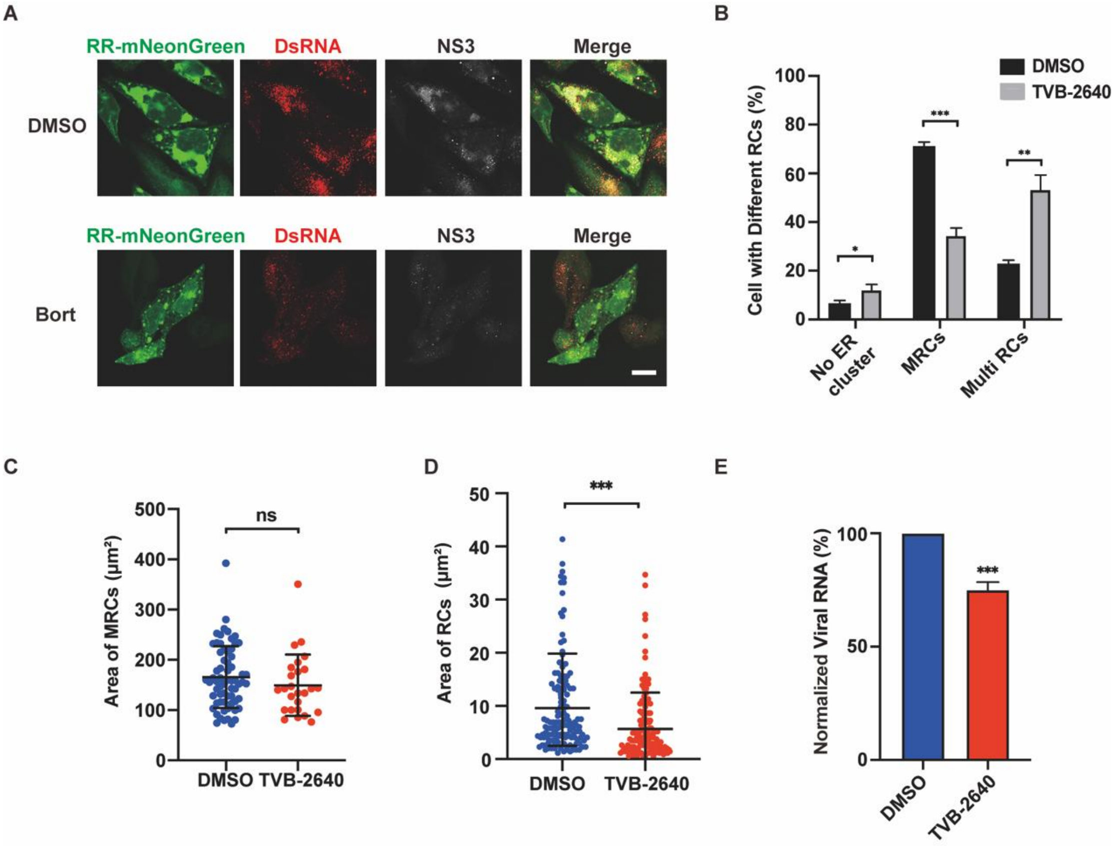

Figure S6 Formation of MRCs is important for viral replication.

(A) Bortezomib (20 nM) treatment impaired MRCs formation and significantly decreased viral dsRNA. Scale bar, 20μm.

(B) The percentage of ZIKV-infected cells with different types of RCs upon the treatment of TVB-2640. More than 200 ZIKV-infected cells were calculated according to the type of viral RCs.

(C) Area of MRCs were measured and plotted upon the treatment of DMSO or TVB-2640 (1 μM).

(D) Area of RCs were measured and plotted upon the treatment of DMSO or TVB-2640 (1 μM).

(E) TVB-2640 (1 μM) inhibited ZIKV replication. Total RNA was extracted from DMSO- or TVB-2640-treated ZIKV-infected cells and the amount of viral RNA was determined by RT-qPCR.

Data are presented as mean ± SD. The P values are obtained from two-tailed t-test, * $p < 0.05$, **$p < 0.01$, ***$p < 0.001$. ns, not significant.

Videos S1-8

Video S1, 3D structure of regular ER networks of HeLa cells

Video S2, 3D structure of ER networks of ZIKV-infected HeLa cells

Video S3, ER dynamics after ZIKV infection (starting time is ~16 hpi)

Video S4, The growth of ZIKV RC α-ε

Video S5, The growth of ZIKV RC γ

Video S6, The growth of ZIKV RC δ

Video S7, The merging of ZIKV RC I

Video S8, The merging of ZIKV RC II
